# Diurnal Activity Patterns of *Elaeidobius* Pollinators on Oil Palm Female Inflorescences in Côte d’Ivoire

**DOI:** 10.3390/insects17060571

**Published:** 2026-05-30

**Authors:** Malanno Kouakou, N’klo Hala, Hauverset Assiénin N’guessan

**Affiliations:** National Agricultural Research Center of Côte d’Ivoire (CNRA), Abidjan 01 BP 1740, Côte d’Ivoire; nklo.hala@cnra.ci (N.H.); hauverset.nguessan@cnra.ci (H.A.N.)

**Keywords:** oil palm, insect pollinator, *Elaeidobius kamerunicus*, *Elaeidobius plagiatus*, *Elaeidobius singularis*, *Elaeidobius subvittatus*, Côte d’Ivoire

## Abstract

Oil palm (*Elaeis guineensis* Jacq.) production in Côte d’Ivoire depends on insect-mediated pollination by *Elaeidobius* weevils. From October 2011 to November 2013, we monitored 432 female inflorescences in three major production zones (La Mé, Grand Béréby, and Iboké). Each month, visits by *E. kamerunicus*, *E. plagiatus*, *E. singularis*, and *E. subvittatus* were recorded on the day of full anthesis during three discrete time periods (09:00–10:00, 11:00–12:00, and 16:00–17:00). Temperature and relative humidity were measured concurrently. Visitation peaked at 11:00 across sites, with abundance ~4-fold higher than at 09:00 and ~20-fold higher than at 16:00; mixed-model results indicated that this temporal pattern was consistent among sites (non-significant Site × Time interaction). *E. subvittatus* dominated visits to female inflorescences (71–74% of individuals), whereas *E. kamerunicus* showed lower visitation rates. Pollinator abundance was positively correlated with temperature (ρ = 0.18) and negatively correlated with relative humidity (ρ = −0.13), although these relationships were weak. These results identify late morning as a key activity period within the observation windows and suggest that adjusting field practices (e.g., insecticide timing) may reduce non-target impacts on pollinators.

## Simple Summary

Oil palm production in Côte d’Ivoire relies on insect pollination by *Elaeidobius* weevils. We monitored visits to female inflorescences in three major production zones (La Mé, Grand-Béréby, and Iboké) during three standardized time windows (09:00–10:00, 11:00–12:00, and 16:00–17:00). Pollinator activity showed a strong late-morning peak at 11:00, with about four times more weevils than at 09:00 and about twenty times more than at 16:00; this pattern was consistent across sites. These findings identify late morning as the key period for pollen transfer within the sampled windows and suggest that avoiding insecticide applications around 11:00 could help protect pollinators and support sustainable oil palm management.

## 1. Introduction

Insect pollinators are essential for the reproduction of many tropical crops by ensuring pollen transfer and fertilization. In oil palm (*Elaeis guineensis* Jacq.), pollination is largely insect-mediated, making pollinator abundance and behavior key determinants of fruit set and yield [1,2,3]. In Côte d’Ivoire, oil palm pollination is mainly performed by Curculionidae weevils of the genus *Elaeidobius*, notably *E. kamerunicus*, *E. plagiatus*, *E. singularis*, and *E. subvittatus* [4]. Although these insects are closely associated with male inflorescences (their feeding and breeding sites), visits to female inflorescences are required for successful pollination and fruit set [5].

Pollinators in agricultural landscapes face growing environmental and anthropogenic pressures. Climate change and pesticide use threaten pollinator populations, and accurately characterizing diel activity patterns is important for effective conservation [6,7]. In oil palm plantations, insecticides can reduce *Elaeidobius* abundance and compromise pollination services [8]. Moreover, microclimatic conditions (temperature and relative humidity) can influence insect activity and flight performance, potentially contributing to within-day variation in visits to female inflorescences [6].

Beyond overall abundance, the timing of pollinator visits is a key component of pollination ecology. Visitation is rarely constant throughout the day and often follows diel patterns shaped by climatic factors and plant-mediated cues [7]. Late-morning peaks in *Elaeidobius* activity have been reported in other oil palm-growing regions [8,9], highlighting the need to identify local peak periods on female inflorescences.

In Côte d’Ivoire, information on *Elaeidobius* activity on female oil palm flowers remains limited, despite its relevance for pollinator-friendly management. This study aimed to characterize *Elaeidobius* visitation to oil palm female inflorescences across three standardized daytime observation windows (09:00–10:00, 11:00–12:00, and 16:00–17:00) in major production zones of Côte d’Ivoire. Specifically, we aimed to: (i) quantify the relative contribution of *Elaeidobius* species visiting female inflorescences; (ii) test whether visitation intensity differs among observation windows and whether the temporal pattern is consistent among sites; and (iii) evaluate associations between temperature, relative humidity, and pollinator abundance. This information may help optimize field practices (particularly insecticide applications) to reduce impacts on pollinators while maintaining pest control efficacy [10].

## 2. Materials and Methods

### 2.1. Study Area

**Figure 1:**
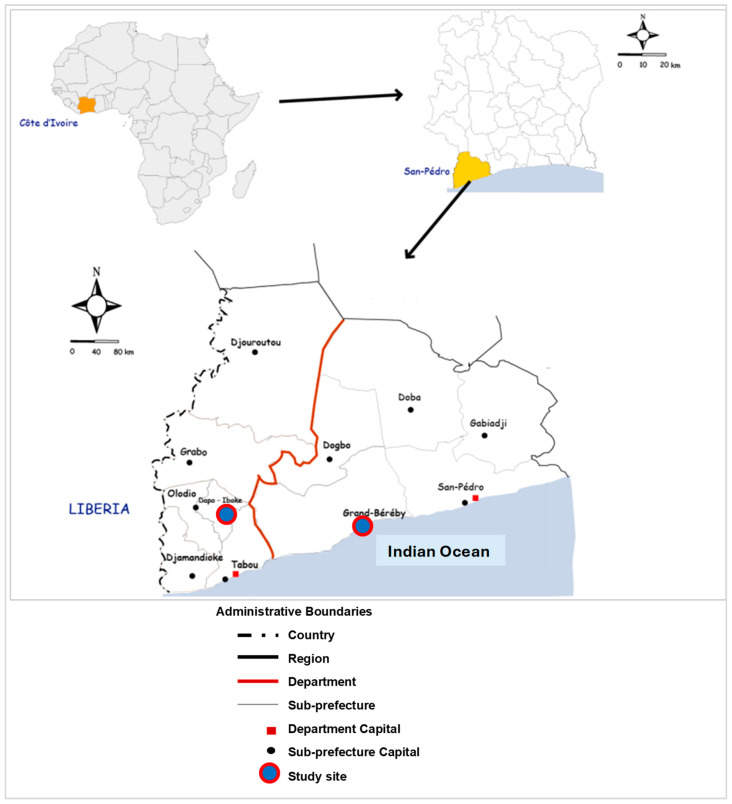
Location of the study sites [13].

This study was conducted in three major oil palm production zones in Côte d’Ivoire: La Mé, Grand-Béréby, and Iboké (Figure 1). La Mé is a research station in southeastern Côte d’Ivoire, ~30 km from Abidjan (5°26′ N, 3°50′ W). The area has a transitional equatorial climate with four seasons; mean annual rainfall is ~1500 mm and mean temperature ranges from 25 to 29 °C [11,12]. Grand-Béréby (5°00′ N, 6°05′ W) and Iboké (4°08′ N, 7°04′ W) are in southwestern Côte d’Ivoire and have a subequatorial climate with two rainy and two dry seasons. Annual rainfall ranges from 1400 to 2400 mm, and temperatures are relatively stable (28–29 °C). Temperatures at these sites range between 25 and 32 °C. Relative humidity varies between 50% and 95% [11].

### 2.2. Study Design

At each site, three plantations were selected (nine plantations in total). Observations were conducted on 5–10-year-old oil palms (*Elaeis guineensis* Jacq.) of the improved C1001 F variety [12]. In each plantation, a central plot of 200 palms was delimited to minimize edge effects. The experimental unit was a female inflorescence at full anthesis.

### 2.3. Field Observations

Once per month, two female inflorescences per plantation (six per site per month) were selected, tagged, and covered with an insect-proof muslin bag to standardize observations and provide a consistent landing surface for incoming pollinators [14].

Target pollinators were Curculionidae weevils of the genus *Elaeidobius* (*E. kamerunicus*, *E. plagiatus*, *E. singularis*, and *E. subvittatus*) [4]. Observations were conducted on the day of full anthesis, when most spikelets are open and receptive. Each bagged inflorescence was monitored during three discrete time periods (09:00–10:00, 11:00–12:00, and 16:00–17:00) selected based on prior studies [14,15,16]. During each period, insects landing on the muslin were counted for 10 min. Individuals were then captured and placed in labeled pill vials containing 70% ethanol. Specimens were identified in the laboratory under a stereomicroscope (Wild Heerbrugg, Heerbrugg, Switzerland) using external morphological characters (e.g., body size, rostrum shape, and elytral coloration pattern), following published identification guides and earlier work on *Elaeidobius* in Côte d’Ivoire and West Africa [4,14,15]. From October 2011 to November 2013, 432 female inflorescences were monitored across all sites. Because observations were restricted to three-time windows, this sampling design may not capture the full diel activity cycle.

These periods represent morning, late morning, and late afternoon activity windows and facilitate comparison with previously reported late-morning peaks in *Elaeidobius* activity [8,9,14].

### 2.4. Microclimatic Measurements

Air temperature (°C) and relative humidity (%) were measured in each plot using a portable thermo-hygrometer. Measurements were taken during each observation period, as close as possible to the monitored inflorescence, to characterize microclimatic conditions at the time of visitation.

### 2.5. Statistical Analysis

The dataset has a hierarchical structure with nested observations (site, plantation, month, and sampling period). To account for non-independence (pseudo-replication), we fitted generalized linear mixed models (GLMMs) with a negative binomial distribution, which is more appropriate than a Poisson distribution for overdispersal count data. Fixed effects included site, sampling period, and their interaction (site × period). Random effects included plantation nested within site and month. Analyses were conducted in R version 4.4.0 [16] using the lme4 package version 1.1-35 [17] and the glmmTMB package version 1.1.9 [18].

In addition to GLMMs, Kruskal–Wallis tests were used to compare abundances among the three sampling periods (09:00, 11:00, and 16:00), followed by Mann–Whitney U post hoc tests with Bonferroni correction (adjusted α = 0.0167). Effect sizes were quantified using Cliff’s delta (Cliff’s δ), with 95% confidence intervals obtained by bootstrap resampling (10,000 iterations). Differences in *Elaeidobius* species composition among sites and sampling periods were tested using PERMANOVA (Bray–Curtis distances) after Hellinger transformation (9999 permutations). Homogeneity of multivariate dispersions was assessed with betadisper. Multivariate analyses were performed using vegan package version 2.8-0 [19]. The proportion of *E. subvittatus* relative to total *Elaeidobius* abundance was analyzed using beta regression (betareg package version 3.2-4) [20,21]. To avoid exact 0 and 1 values, proportions were transformed as (y + ε)/(*n* + 2ε) with ε = 0.001. Site and sampling period were included as predictors.

The potential influence of climatic factors on pollinator abundance was examined by including temperature (T, °C) and relative humidity (RH, %) as covariates in the GLMM. Spearman’s rank correlations were calculated to assess the relationship between climatic variables and *Elaeidobius* abundance.

## 3. Results

### 3.1. Effect of Collection Time on Total Elaeidobius Abundance

**Figure 2:**
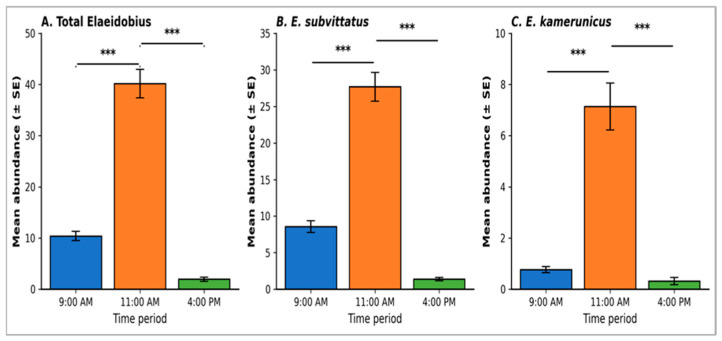
Temporal variation in *Elaeidobius* abundance. (**A**) Total *Elaeidobius*, (**B**) *E. subvittatus*, (**C**) *E. kamerunicus*. Bars represent mean abundance (±SE). *** *p* < 0.001 (Mann–Whitney tests).

*Elaeidobius* abundance showed highly significant variation according to time (H = 513.4; df = 2; *p* < 0.001). A marked activity peak was observed at 11:00 (40.18 ± 2.76 individuals/inflorescence), approximately 4 times higher than at 09:00 and 20 times higher than at 16:00. This pattern was consistent for both dominant species: *E. subvittatus* (H = 443.6; *p* < 0.001) and *E. kamerunicus* (H = 221.1; *p* < 0.001) (Figure 2).

### 3.2. Effect Sizes and Abundance Ratios

Effect sizes (Table 1) confirm the biological significance of the observed differences. The largest effect was found between 11:00 and 16:00 (δ = 0.71, large effect), representing a 20-fold difference in pollinator abundance. The comparison between 11:00 and 09:00 showed a medium-to-large effect (δ = 0.42), with approximately 4 times more individuals at 11:00.

### 3.3. Site Effect and Robustness of the Temporal Pattern

**Figure 3:**
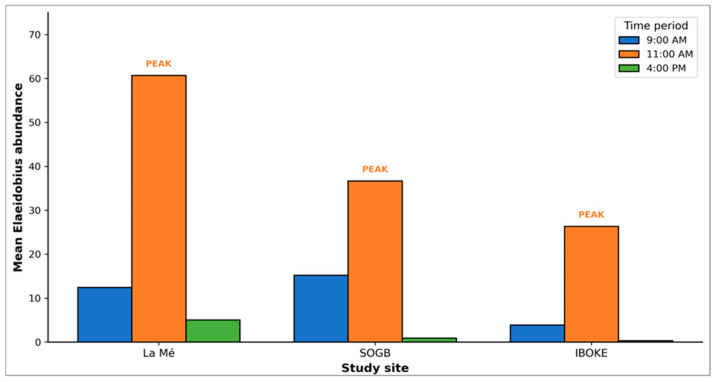
Mean abundance of *Elaeidobius* by site and time.

The GLMM with Negative Binomial distribution confirmed significant effects of both time (*p* < 0.001) and site (*p* < 0.01) after correcting for the hierarchical data structure. La Mé showed the highest overall abundance, followed by Grand-Béréby and Iboké. Importantly, the Site × Time interaction was not significant (*p* > 0.05), indicating that the temporal pattern (peak at 11:00) was consistent across all three study sites, reinforcing the robustness of this finding. This pattern is illustrated in Figure 3.

### 3.4. Species Composition

**Figure 4:**
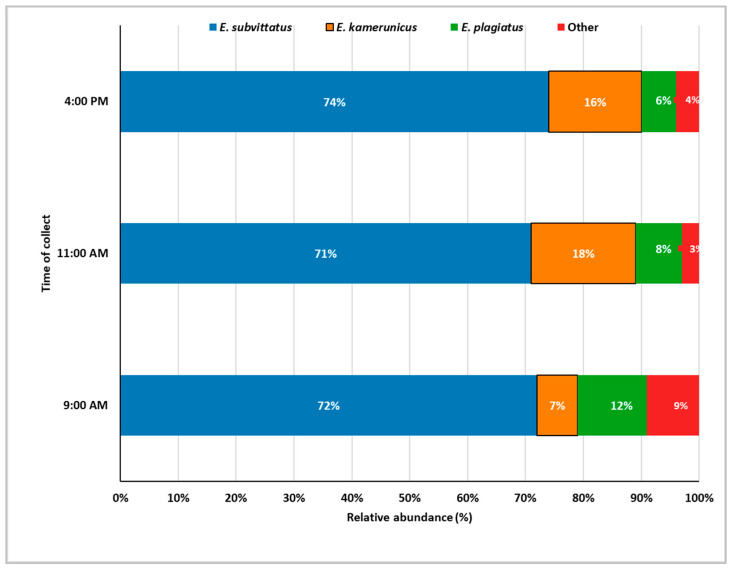
Relative species composition.

PERMANOVA analysis (Table 2) revealed significant effects of both Site (Pseudo-F = 28.7; R^2^ = 3.3%; *p* = 0.001) and Time period (Pseudo-F = 20.6; R^2^ = 2.4%; *p* = 0.001) on the composition of the four *Elaeidobius* species. The non-significant Site × Time interaction (*p* = 0.142) confirms that the species composition pattern across time periods was consistent among study sites. This pattern is also shown in Figure 4.

Multivariate dispersion did not differ detectably among groups (betadisper), supporting the interpretation of PERMANOVA results as differences in centroids rather than heterogeneity of dispersion. In addition, beta regression indicated that the proportion of *E. subvittatus* did not vary significantly among time periods.

### 3.5. Relationship Between Temperature, Relative Humidity, and Elaeidobius Abundance

#### 3.5.1. Descriptive Statistics of Climatic Variables

Temperature ranged from 22.4 to 38.2 °C (mean = 29.3 ± 3.2 °C) and relative humidity from 38.7 to 98.2% (mean = 72.5 ± 12.9%). Climatic conditions varied significantly among time periods: temperature was highest at 11:00 (30.1 ± 2.9 °C) compared to 09:00 (28.1 ± 2.3 °C) and 16:00 (29.8 ± 3.5 °C), while relative humidity was lowest at 11:00 (68.8 ± 11.7%) compared to 09:00 (78.5 ± 12.5%) (Table 3).

#### 3.5.2. Influence of Climatic Factors

**Figure 5:**
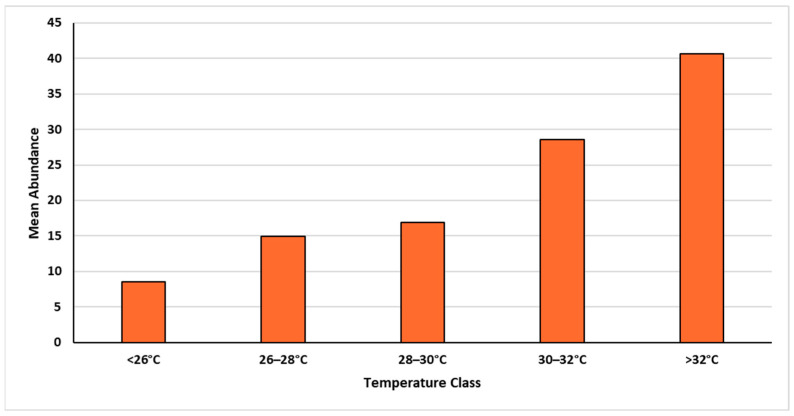
Mean *Elaeidobius* Abundance by Temperature Class.

**Figure 6:**
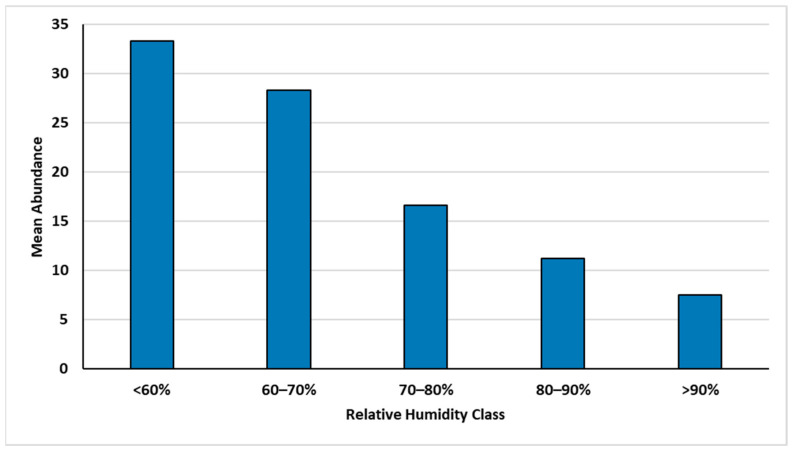
Mean *Elaeidobius* Abundance by Relative Humidity Class.

*Elaeidobius* abundance was positively correlated with temperature (Spearman ρ = 0.18; *p* < 0.001) and negatively correlated with relative humidity (ρ = −0.13; *p* < 0.001) (Table 4). Mean abundance increased from 8.5 individuals per inflorescence at temperatures < 26 °C to 40.6 individuals at temperatures > 32 °C (Figure 5). Similarly, abundance decreased from 33.3 individuals at RH < 60% to 7.5 individuals at RH > 90% (Figure 6).

## 4. Discussion

This study describes *Elaeidobius* visitation to oil palm female inflorescences across three major production zones of Côte d’Ivoire. Across the three standardized observation windows, visitation was strongly time-structured, with a pronounced late-morning peak at 11:00. Abundance at 11:00 was ~4-fold higher than at 09:00 and ~20-fold higher than at 16:00, with medium-to-large to large effect sizes (Cliff’s δ = 0.42 and 0.71, respectively). Importantly, the Site × Time interaction was not significant in the GLMM, indicating that the 11:00 peak was consistent across sites and therefore represents a robust feature of pollinator activity within the windows sampled. Similar late-morning peaks have been reported in other oil palm-growing regions [9,16], suggesting that this temporal pattern may be widespread.

Taken together, these results suggest that late morning is the period of greatest potential pollen transfer to receptive female inflorescences in Côte d’Ivoire. This inference is based on visitation intensity; confirming consequences for reproductive success will require linking visitation to pollen loads on weevils and to fruit set.

Pollinators were monitored in three discrete 1-h windows (09:00–10:00, 11:00–12:00, and 16:00–17:00). Consequently, our data do not provide a continuous description of activity across the full diel cycle and cannot rule out additional peaks (e.g., early morning, mid-afternoon, or near dusk). Conclusions should therefore be interpreted as evidence of a late-morning peak within the windows sampled. Future studies should increase within-day resolution (additional time points and/or continuous sampling) and test whether peak timing varies with season, flowering phenology, or local management [6,15].

The late-morning increase in visitation coincided with warmer and less humid conditions. In our data, total *Elaeidobius* abundance was positively correlated with temperature (Spearman ρ = 0.18) and negatively correlated with relative humidity (ρ = −0.13), and mean abundance increased sharply at higher temperature classes (>32 °C) while decreasing at high humidity (>90%). Although statistically significant, these correlations were weak, indicating that microclimate is only one component of within-day visitation dynamics. We therefore hypothesize that additional biological drivers contribute to the timing and magnitude of visits, including (i) local availability of male inflorescences as feeding/breeding resources, which may modulate daily movement between male and female inflorescences; and (ii) plant-mediated cues that vary across the day and influence attraction and orientation [6,7]. These mechanisms were not directly measured in this study and should be tested explicitly in future work.

Beyond time-of-day effects, the GLMM also detected differences in overall abundance among sites, with La Mé showing higher visitation than the southwestern sites. Because the temporal peak was conserved across sites (non-significant Site × Time interaction), site differences likely reflect broader determinants of population size rather than differences in diel timing. Potential drivers include local availability of male inflorescences (breeding/feeding resources), plantation structure, and phytosanitary practices [14,15]. Insecticide applications can reduce *Elaeidobius* abundance under some management conditions [10], and contrasts between industrial and village plantations may contribute to spatial variation. These factors were not quantified here and should be addressed in future studies.

*Elaeidobius* weevils are primarily associated with male inflorescences (feeding and breeding sites), yet visits to female inflorescences are required for pollen transfer. Besides weather, plant-mediated cues may shape movement and within-day visitation. Diel variation in attractant signals (including volatile emissions) has been proposed as a driver of time-specific visitation in oil palm and is discussed in broader syntheses of *Elaeidobius*-mediated pollination [6,10]. Given that we did not measure volatile emissions, male inflorescence availability, or weevil pollen loads, we do not interpret these mechanisms as demonstrated here. Instead, we propose them as working hypotheses to explain why the 11:00 peak was consistent across sites: (i) microclimatic thresholds that permit flight and activity; (ii) synchronized plant cues that increase attraction to receptive female inflorescences; and/or (iii) synchronized emergence or dispersal from male inflorescences. Testing these hypotheses will require concurrent measurements of floral resources, chemical emissions, and weevil condition/loads across finer time intervals.

Species composition analyses indicated that the assemblage varied with site and time, yet the Site × Time interaction was not significant, supporting a broadly similar temporal shift in composition across sites. Across all sites and time periods, *E. subvittatus* dominated captures on female inflorescences (≈71–74% of individuals), and beta regression detected no effect of time on its proportion. Thus, while absolute abundance changed dramatically through the day, relative dominance remained stable, suggesting that the different *Elaeidobius* species respond in parallel to the within-day activity cycle.

The consistent predominance of *E. subvittatus* on female inflorescences contrasts with the frequent emphasis on *E. kamerunicus* as a key oil palm pollinator in West Africa, partly because of its documented links with fruit set and pollen transfer efficiency [2,3,5]. In our study, *E. kamerunicus* was less abundant than *E. subvittatus*, but abundance alone does not determine pollination effectiveness. Species may differ in pollen load, behavior on receptive spikelets, and dispersal between male and female inflorescences [15]. Quantifying pollen loads and relating species-specific visitation to fruit set would therefore be necessary to assess whether the observed assemblage has implications for pollination services in Côte d’Ivoire.

From an applied perspective, identifying late morning as the period of highest visitation within our sampling windows has implications for plantation management. Insecticide applications during peak visitation may disproportionately affect pollinators and reduce pollination services, as previously reported for oil palm systems. Shifting interventions away from peak visitation (e.g., early morning or late afternoon) may reduce non-target impacts while maintaining pest control efficacy [10]. Overall, integrating within-day pollinator activity patterns into management decisions may support more sustainable production while safeguarding pollination services.

## 5. Conclusions

Within the three observation windows, *Elaeidobius* visitation to oil palm female inflorescences showed a consistent late-morning peak at 11:00 across all study sites in Côte d’Ivoire. Visitation was associated with warmer and less humid conditions, although correlations with temperature and relative humidity were weak. Across sites and time periods, *E. subvittatus* was the dominant species on female inflorescences. These findings support pollinator-friendly management, particularly by avoiding insecticide applications during peak visitation. Future work should broaden within-day sampling and link visitation and species-specific activity to pollen transfer and fruit set. Overall, integrating within-day pollinator activity patterns into management decisions may support more sustainable production while safeguarding pollination services.

## Figures and Tables

**Table 1:** Effect sizes (Cliff’s δ) and abundance ratios between time periods.

Comparison	Cliff′s δ	95% CI	Magnitude	Abundance Ratio
11:00 vs. 09:00	0.42	[0.37, 0.47]	Medium–Large	3.87 [3.21, 4.68]
11:00 vs. 16:00	0.71	[0.66, 0.76]	Large	20.5 [15.8, 27.2]
09:00 vs. 16:00	0.38	[0.32, 0.44]	Medium	5.30 [4.12, 6.91]

CI = Confidence Interval (bootstrap, 10,000 replications); Magnitude thresholds: |δ| < 0.147 negligible; 0.147–0.33 small; 0.33–0.474 medium; >0.474 large.

**Table 2:** PERMANOVA results on *Elaeidobius* species composition (Bray–Curtis distance).

Source of Variation	df	Sum Sq	Pseudo-F	R^2^	Pr (>F)
Site	2	12.45	28.7	3.30	0.001 ***
Time	2	8.92	20.6	2.40	0.001 ***
Site:Time	4	1.23	1.4	0.30	0.142 ns
Residual	1618	350.2	-	93.90	-
Total	1626	372.8	-	100	-

Hellinger-transformed abundance data; 9999 permutations, *** *p* < 0.001; ns = not significant.

**Table 3:** Climatic conditions by time.

Time Period	Temperature (°C)	Relative Humidity (%)
09:00	28.1 ± 0.10	76.1 ± 0.53
11:00	29.7 ± 0.12	68.8 ± 0.50
16:00	29.4 ± 0.15	68.9 ± 0.52
Kruskal–Wallis H	45.2	89.4
*p*	<0.001	<0.001

**Table 4:** Correlation between climatic factors and abundance.

Factor	Correlation (rho)	Significance (*p*)
Temperature	0.18	<0.001
Relative Humidity	−0.13	<0.001
T × RH	−0.45	<0.001

## Data Availability

The raw data supporting the conclusions of this article will be made available by the authors on request.
